# Channel Confinement of Aromatic Petrochemicals via Aryl–Perfluoroaryl Interactions With a B←N Host

**DOI:** 10.3389/fchem.2019.00695

**Published:** 2019-10-22

**Authors:** Gonzalo Campillo-Alvarado, Megan M. D'mello, Michael A. Sinnwell, Herbert Höpfl, Hugo Morales-Rojas, Leonard R. MacGillivray

**Affiliations:** ^1^Department of Chemistry, University of Iowa, Iowa City, IA, United States; ^2^Centro de Investigaciones Químicas, Instituto de Investigación en Ciencias Básicas y Aplicadas, Universidad Autónoma del Estado de Morelos, Cuernavaca, Mexico

**Keywords:** boron, boronic acids, boronic esters, self-assembly, host-guest chemistry, crystal engineering, inclusion chemistry, channel confinement

## Abstract

We report channel confinement properties of an electron-deficient boron host derived from the orthogonal B←N interaction between a boronic ester and *trans*-pentafluorostilbazole. The boron host forms one-dimensional channels in the crystalline solid state when crystallized with common electron-rich aromatic petrochemicals (i.e., benzene, toluene, *o*-xylene) to form solvates and a cocrystal with stilbene. Molecular confinement of the electron-rich molecules in the solids is achieved through a combination of aryl–perfluoroaryl interactions (π-π_F_) and hydrogen bonds.

## Introduction

Host–guest chemistry is widely considered a landmark of supramolecular chemistry and focuses on uses of non-covalent interactions to hold together multicomponent molecular aggregates (Steed and Atwood, 2013; Xiao et al., 2019a,b). In the context of crystal engineering, hosts that provide the ability to confine guests into channel-type architectures have received increased attention due to intriguing properties (e.g., catalysis Yang et al., 2006, dynamics Wu et al., 2019, photoconduction Quintel and Hulliger, 1999, sorption Lim et al., 2008, and separation Chen et al., 2013; Barton et al., 2019). Channel formation in transmembrane ionic transport is a vital process for living cells (Haynes and Gale, 2011). However, in contrast to the large number of natural and synthetic inorganic zeolites (Ramamurthy and Eaton, 1994; Tabacchi, 2018), there is a significant lower number of purely-organic molecules identified as reliable channel-formers in closed-packed systems (e.g., calixarene, phenylacetylene, tetraphenylethylene, triazine building blocks) (Moore, 1997; Langley and Hulliger, 1999; Dalgarno et al., 2007; Couderc and Hulliger, 2010; He et al., 2011; Huang et al., 2019; Lin et al., 2019). Understanding the formation of open cavities or channels in organic systems is essential for the development of extended systems such as Hydrogen-Bonded Organic Frameworks (HOFs) (Helzy et al., 2016; Karmakar et al., 2016) and Covalent Organic Frameworks (COFs) (Feng et al., 2012; Xu et al., 2016).

In this context, an emerging solid-state ordering strategy relies on the use of boronic acids and derivatives to confine guests into a crystal lattice (Nishiyabu et al., 2011; Bull et al., 2013). The inclusion properties are facilitated by multiple supramolecular interactions of boronic acids and derivatives (e.g., hydrogen bonding (Campillo-Alvarado et al., 2018a,b; Ruelas-Alvarez et al., 2019), reversible esterification Fornasari et al., 2019; Takahashi et al., 2019, π-π interactions and B←N coordination Campillo-Alvarado et al., 2019; Ono and Hisaeda, 2019; Stephens et al., 2019).

**Scheme 1:**
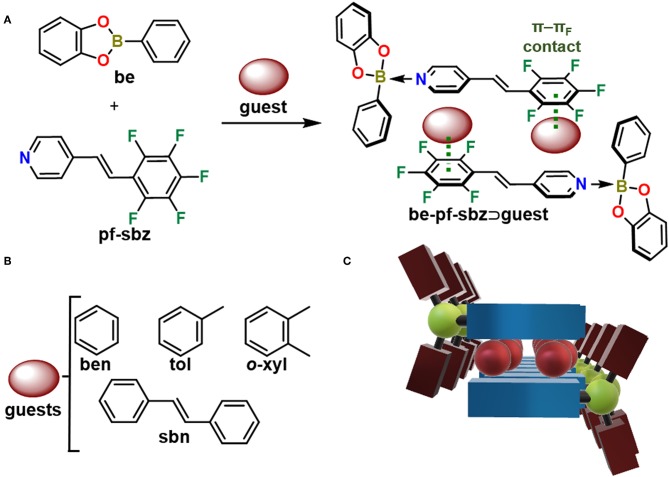
**(A)** Self-assembly of **be-pf-sbz**⊃**guest** from **be** and **pf-sbz** in the presence of a guest. **(B)** Aromatic guests in this study. **(C)** Formation of channels in the solid state.

As part of our efforts to investigate boron-based host materials (Herrera-España et al., 2015; Campillo-Alvarado et al., 2018c), we report the synthesis and channel confinement properties of a highly electron-deficient adduct (**be-pf-sbz**) (Scheme 1A). The boron host **be-pf-sbz** is primarily sustained by a B←N bond between phenylboronic acid catechol ester (**be**) and *trans*-pentafluorostilbazole (**pf-sbz**). The purpose of this work is to evaluate solid-state confinement of π-electron-rich molecules commonly employed in the petrochemical industry [benzene (**ben**), toluene (**tol**), *o*-xylene, (***o*-xyl**)] (Scheme 1B) using **be-pf-sbz**. The alkene stilbene (**sbn**) is also studied as a guest. Aryl–perfluoroaryl interactions (π-π_F_) (Coates et al., 1998; Sinnwell et al., 2015; Martínez-Vargas et al., 2017) and hydrogen bonds assist the confinement of petrochemicals into electron-deficient channels (Scheme 1C). To the best of our knowledge, our work represents the first example of a discrete B←N adduct that consistently generates channel-type architectures in the solid state upon self-assembly with guests. A related and previous example of organoboron channel former employs the hydrogen bonding capacity of tetraboronic acids to achieve channel-type architectures (Fournier et al., 2003).

## Results and Discussion

### Generation of Solvate-Based Channels

Our strategy to form host-guest materials involves the coordination of **be** and **pf-sbz** to generate an electron-deficient adduct. The boron adduct would then enable the confinement of π-rich aromatic guests through π-π_F_ interactions.

To test our general hypothesis, **be** (30 mg, 0.1530 mmol) was added to a vial containing **pf-sbz** (41.5 mg, 0.1530 mmol) in **ben** (2 mL). The vial was heated until the solution adopted a clear red coloration. Orange single crystals formed as plates after 2 days of slow evaporation (see Supplementary Material for additional experimental information). ^1^H NMR spectroscopy revealed the composition of crystals to be **be-pf-sbz** ⊃**ben** (see Supplementary Figure 1 for 1H NMR of single crystals).

**Figure 1:**
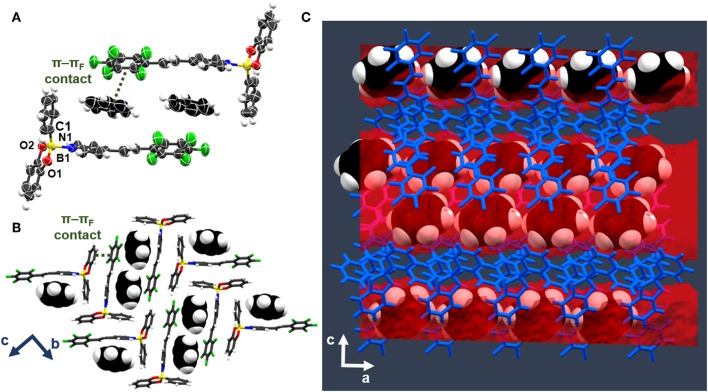
X-ray structure be-pf-sbz⊃ben: **(A)** stacking involving T-shaped adducts be-pf-sbz⊃ben, **(B)** p-pF, C-H···p, and van der Waals interactions in bc-plane, and **(C)** channel formation along a-axis.

A scXRD analysis of **be-pf-sbz** ⊃**ben** demonstrates the components of the solid to crystallize in the monoclinic space group *P*2_1_/*n* (see Supplementary Table 1 for crystallographic parameters). The asymmetric units consist of a molecule of both **be-pf-sbz** and **ben** (Figure 1). The stilbazole **pf-sbz** is coordinated to **be** through a B←N bond [1.674(3) Å] forming a discrete T-shaped adduct. The tetrahedral character of boron (*THC* = 71.7%) (Höpfl, 1999) shows the strength of the B←N bond to be comparable to similar adducts (Cruz-Huerta et al., 2016; Campillo-Alvarado et al., 2019). The fluorinated alkene adopts a twisted conformation (29.3°), while the pyridyl ring lies approximately orthogonal (89.7°) to the best plane of atoms C1, O1, and O2. Importantly, a **ben** molecule resides within one-dimensional (1D) channels along *a*, being sustained though face-to-face π-π_F_ interactions with the perfluoroaryl ring of **be-pf-sbz** (centroid···centroid = 3.745 Å). The channels are defined by adjacent complexes in such a way that a cavity containing two **ben** molecules related by an inversion center is formed (Figure 1A). The **ben** guests occupy 25.1% (i.e., contact surface) of the unit cell volume and are distributed within the channels that run along the *a*-axis (Figure 1B). The channels are further sustained by C–H···π interactions and van der Waals contacts from **ben** and the aryl ring of an adjacent **be-pf-sbz** molecule (Figure 1C).

**Figure 2:**
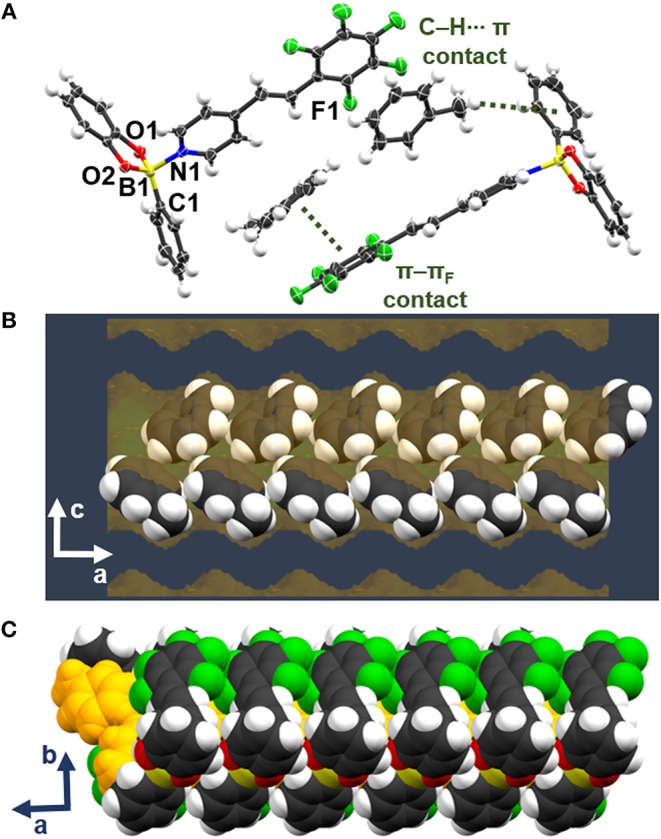
X-ray structure **be-pf-sbz**⊃**tol**: **(A)** stacking involving T-shaped adducts **be-pf-sbz**⊃**tol**, **(B)** corrugated **tol** stacks along the *a*-axis, and **(C)** space-filling view of channel confinement.

Generality of the channel forming properties of **be-pf-sbz** was confirmed when **tol** and ***o-*xyl** were used as crystallization solvents. Both solvents were confined in the crystal lattice as observed by scXRD analysis of single crystals (orange prisms) (Figure 2) and shown indirectly by ^1^H NMR spectroscopy.

Specifically, scXRD analysis of **be-pf-sbz**⊃**tol** and **be-pf-sbz**⊃***o*-xyl** revealed the solids to be structurally different from **be-pf-sbz**⊃**ben**, but isostructural among themselves, crystallizing in the chiral orthorhombic space group *P*2_1_2_1_2_1_. The asymmetric units consist of a molecule of both **be-pf-sbz** and solvent (either **tol** or ***o*-xyl**), effectively being defined as pseudopolymorphs (Nangia, 2006) (Figure 2A). The B←N bond distances [1.685(5) Å and 1.680(3) Å] and *THC*s (70.1° and 71.1°) of **be-pf-sbz**⊃**tol** and **be-pf-sbz**⊃***o*-xyl**, respectively, are comparable to **be-pf-sbz**⊃**ben**. While the solvent molecules in **be-pf-sbz**⊃**ben** lie in parallel planes within channels, **tol** and ***o*-xyl** are tilted among each other in neighboring molecular strands (44.2° and 52.7°, respectively) along the *a*-axis (Figures 2B,C). The stilbazole adopts a nearly coplanar conformation (4.0° and 2.6°), with the pyridyl rings being nearly orthogonal (81.7° and 80.3°) to the plane C1, O1, and O2 of the boron-adduct in **be-pf-sbz**⊃**tol** and **be-pf-sbz**⊃**o-xyl**, respectively. The π-π_F_ face-to-face interactions of **be-pf-sbz**⊃**tol** and **be-pf-sbz**⊃***o*-xyl** (centroid···centroid = 3.790 and 4.129 Å, respectively) are weaker than in **be-pf-sbz**⊃**ben** (3.745 Å). The overall host-guest conformations presumably maximize C–H···π interactions with the included solvent. The **tol** and ***o*-xyl** guests effectively occupy 25.5 and 27.4%, which is in agreement with the higher molecular masses of the solvents and host-to-solvent ratio.

### Generation of Cocrystal-Based Channels

The integration of a solid as a guest with **be-pf-sbz** was realized using **sbn** as the coformer.

Specifically, **be** (30 mg, 0.153 mmol) and **pf-sbz** (41.5 mg, 0.153 mmol) were dissolved in a solution of acetonitrile (2 mL) containing **sbn** (13.79 mg, 0.077 mmol). The vial was heated until the solution adopted a clear red coloration. Single crystals of **be-pf-sbz**⊃**sbn** in the form of orange plates were observed after 2 days of slow evaporation.

**Figure 3:**
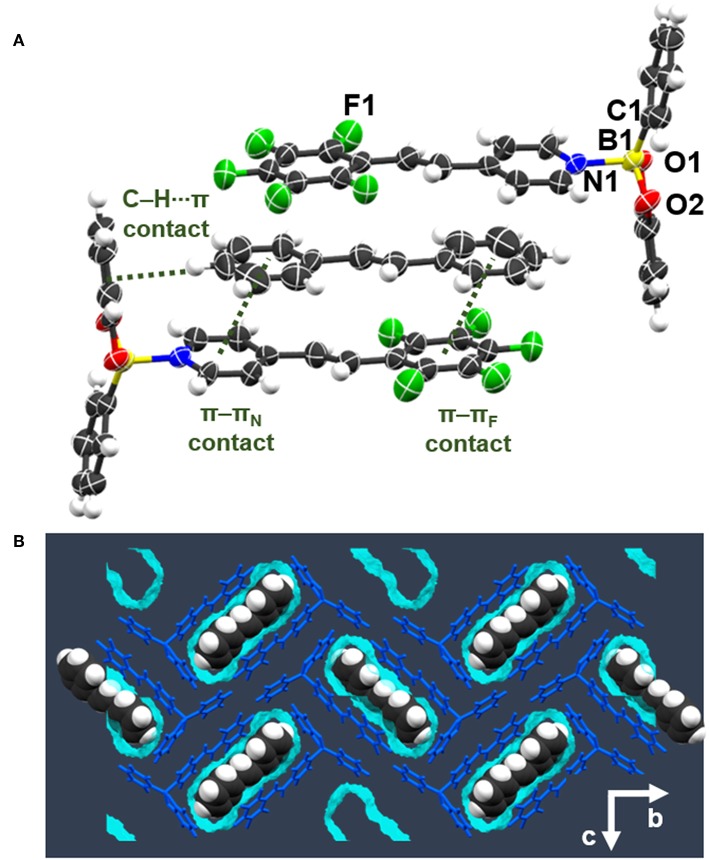
X-ray structure **be-pf-sbz**⊃**sbn**: **(A)** stacking involving T-shaped adducts **be-pf-sbz**⊃**sbn**, **(B)** space-fill view of channel confinement along the *a*-axis.

A scXRD analysis revealed the components of **be-pf-sbz**⊃**sbn** to crystalize in the monoclinic space group *P*2_1_/*n* (Figure 3) (see Supplementary Table 2 for crystallographic parameters). The asymmetric unit consists of one molecule of **be-pf-sbz** and one-half molecule of **sbn**. The larger B←N bond distance (1.802(4) Å) and smaller *THC* (64.8°) of **be-pf-sbz**⊃**sbn** is indicative of the channel confinement of the rigid guest **sbn** to result in a weaker B←N interaction (Höpfl, 1999). The coplanarity (8.6°) and orthogonality (89.5°) of the host effectively maximize the π-π_F_ interactions with **sbn** (centroid···centroid = 4.013 Å) and establish additional π-π_N_ contacts (centroid···centroid = 4.110 Å). Thus, **sbn** acts in turn as a coplanar channel “template” (Langley and Hulliger, 1999) (Figure 3A). Additional edge-to-face C–H···π interactions of **be-pf-sbz** sustain **sbn** in channels along the *a*-axis (Figure 3B). Although the overall packing is close to being isostructural with **be-pf-sbz**⊃**ben** [**sbn** occupies the same unit cell volume (25.1%)], the total cell volume increases by approximately 160 A^3^, in agreement with the higher molecular mass of **sbn**.

### Generation of Apohost

When *m*- and *p*-xylene xylenes were used as crystallization solvents, the formation of prohost **be-pf-sbz** was observed vs. a solvate. Single crystals in the form of pale-yellow plates of pure **be-pf-sbz** were obtained by slow evaporation of a *p*-xylene solution (2 mL) of **be** (30 mg, 0.153 mmol) and **pf-sbz** (41.5 mg, 0.153 mmol).

**Figure 4:**
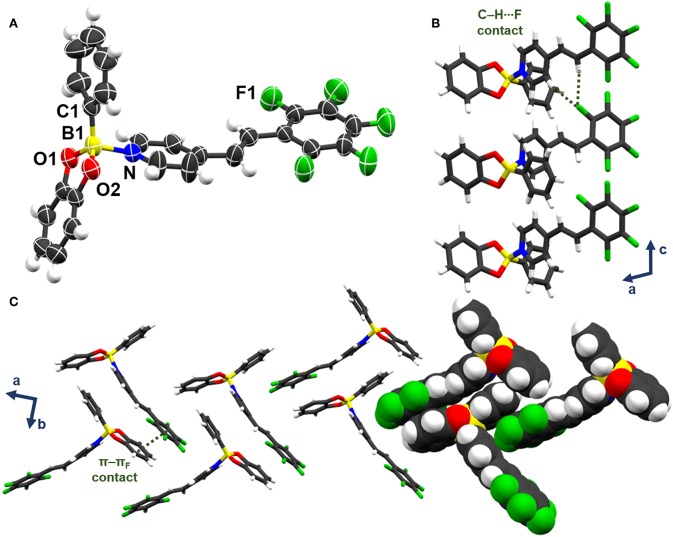
X-ray structure **be-pf-sbz**: **(A)** T-shaped adduct of **be-pf-sbz**, **(B)** chains along the *c*-axis with C–H···F contacts, **(C)** space-fill view of herringbone arrangement in the *ab*-plane.

A scXRD analysis revealed **be-pf-sbz** to crystallize in the monoclinic space group *P*2_1_/*c* with a single molecule of **be-pf-sbz** in the asymmetric unit (Figure 4). The B←N bond [1.678 (4) Å] and *THC* (71%) are similar to **be-pf-sbz** solvates. The fluorinated alkene exhibits a twisted conformation (16.6°) less orthogonal (84.4°) to C1, O1, and O2 of **be** vs. the solvates and cocrystal (Figure 4A). Notably, **be-pf-sbz** molecules display a herringbone arrangement primarily sustained by π-π_F_ interactions (centroid··· centroid = 3.676 Å) between the electron-deficient region of (i.e., fluorinated alkene) and the catecholate motif of an adjacent molecule. Bifurcated C–H···F contacts further form chains along the *c*-axis (Figures 4B,C). The presence of destabilizing C–F···π contacts (Vangala et al., 2002) is also observed. The self-assembly behavior of **be-pf-sbz** in the presence of *m*- and *p*-xylenes likely reflects inadequate surface area of the guests (Swift et al., 1998; Couderc and Hulliger, 2010), which effectively prevents the formation of a crystalline channel architecture. Hartree-Fock calculations (3-21G basis set) of the three xylene isomers revealed ***o*-xyl** to be effectively more compact (surface area: 146.2 Å^2^, volume: 134.3 Å^3^) than *m*- and *p*-xylenes (surface areas: 149.4 and 149.6 Å^2^, volumes: 134.7 and 134.8 Å^3^, respectively) owing to the shorter separation between the methyl groups.

### Inclusion Behavior: Complementarity and Conformational Flexibility

**Figure 5:**
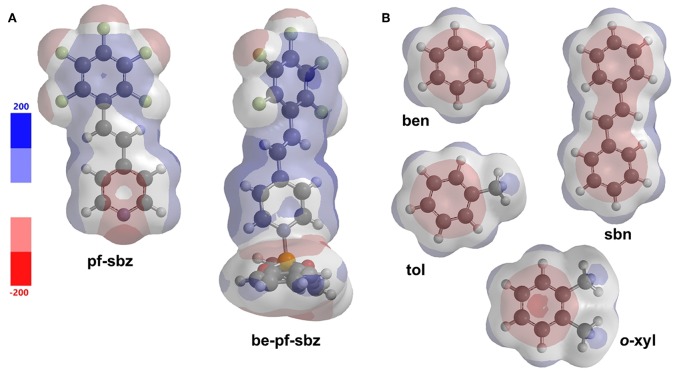
MEP surfaces: **(A)** host and **pf-sbz**, and **(B)** guests.

To shed further light on the inclusion behavior of **be-pf-sbz**, Hartree-Fock calculations (3-21G basis set) were performed using the data from the X-ray experiments (Figure 5). Molecular electrostatic potential (MEP) surfaces revealed **pf-sbz** to be composed of a combination of relatively electron-rich (pyridyl) and electron-deficient (F-arene) rings (i.e., polarized charge distribution). Upon coordination with **be**, both rings are electron-deficient and generate two potential aromatic recognition sites (Wakamiya et al., 2006) (Figure 5A). The inclusion behavior displayed by **be-pf-sbz** can, thus, be attributed to the coordination to the B-atom having triggered the interactions with the electron-rich guests (Figure 5B). We note that the addition of fluorine to organic molecules has been exploited to achieve formation of inclusion complexes (Reichenbächer et al., 2005; Berger et al., 2011).

The inclusion behavior of **be-pf-sbz** contrasts B←N adducts with bipyridines. The ditopic B←N adducts generate completely enclosed cavities vs. channels owing to the presence of edge-to-face π···π interactions with additional boronic esters (Campillo-Alvarado et al., 2018c, 2019). The generation of porous extended frameworks based on the B←N interactions has been recently explored (Cruz-Huerta et al., 2012; Stephens et al., 2019).

**Figure 6:**
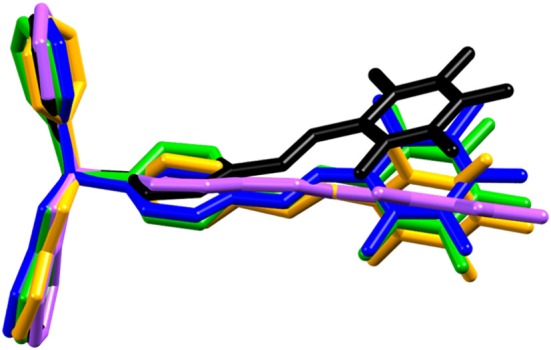
Molecular overlay of **be-pf-sbz** in the X-ray structures of: **be-pf-sbz** (black), **be-pf-sbz**⊃**ben** (blue), **be-pf-sbz**⊃***o*-xyl** (green), **be-pf-sbz**⊃**tol** (orange), **be-pf-sbz**⊃**sbn** (pink).

The diversity of the included guests can be attributed to the conformational flexibility of **be-pf-sbz**. The supramolecular allosteric nature of the host is evidenced by an overlay of the X-ray molecular structures of **be-pf-sbz** (Figure 6) from the five solids. Changes in twist angle of the fluorinated alkene [2.6° (**be-pf-sbz**⊃***o*-xyl**)−29.3° (**be-pf-sbz**⊃**ben**)] effectively serve to optimize π-π_F_ interactions with guests while the boronate ester moiety acts as both a stator and “hinge” by providing additional sites for C–H···π and van der Waals interactions (see Supplementary Table 3 for selected supramolecular interactions of crystals). The observed significant contribution of the guest to the crystal packing of **be-pf-sbz** is reminiscent of the design of inorganic zeolites and other nanostructured materials (Davis et al., 1996; Holman et al., 2001). Indeed, the flexibility allows the host to “shrink-wrap” guests of appropriate size and geometry (Holman et al., 2001).

## Conclusion

In summary, we have demonstrated that a fluorinated boron host (i.e., **be-pf-sbz**) supports the formation of electron-deficient channels in the solid state when crystallized with electron-rich aromatic petrochemicals (i.e., **ben**, **tol**, and ***o*-xyl**) and **sbn**. The persistent channels in host-guest structures are sustained by a combination of face-to-face π-π_F_ and C–H···π interactions. When *m*- and *p*-xylenes are used as crystallization solvents, the formation of the apohost is observed. Current efforts are underway to exploit the confinement properties of **be-pf-sbz** to generate storage and separation materials, and to achieve topochemical control in the solid state of guest.

## Data Availability Statement

All datasets generated for this study are included in the article/Supplementary Files.

## Author Contributions

GC-A carried out experimental work, data analysis, and writing of the original draft. MD'm and MS carried out experimental work. HH and HM-R made intellectual contributions. LM participated as the project administrator and writing of the original draft.

### Conflict of Interest

The authors declare that the research was conducted in the absence of any commercial or financial relationships that could be construed as a potential conflict of interest.

## Supplementary Material

Experimental details and methods, and additional XRD data can be found in the Supplementary Material. X-ray crystallographic information files (CIF) are available for compounds **be-pf-sbz**, **be-pf-sbz**⊃**ben**, **be**-**pf-sbz**⊃**tol**, **be**-**pf-sbz**⊃***o*-xyl**, and **be**-**pf-sbz**⊃**sbn** (CCDC 1943956-1943960).
