# Supplementary material for: Channel Confinement of Aromatic Petrochemicals via Aryl–Perfluoroaryl Interactions With a B←N Host
Source: Front Chem. 2019 Oct 22;7:695. doi: 10.3389/fchem.2019.00695 (PMC6818625; doi:10.3389/fchem.2019.00695)
Supplement: Supplementary file 1 [file Table_1.DOCX]

Supplementary Material

**Channel Confinement of Aromatic Petrochemicals via Aryl−Perfluoroaryl Interactions with a B←N Host**

Gonzalo Campillo-Alvarado,^a^ Megan M. D’mello,^a^ Michael A. Sinnwell,^a^ Hugo Morales-Rojas, Herbert Höpfl,^b^ Leonard R. MacGillivray^a,^*

^a^Department of Chemistry, University of Iowa, Iowa City, IA, 52242-1294, USA.

^b^Centro de Investigaciones Químicas, Universidad Autónoma del Estado de Morelos, Av. Universidad 1001, 62209, Cuernavaca, México.

*e-mail: len-macgillivray@uiowa.edu

# Experimental information

**Materials**. All the reagents and solvents used for this study were purchased from Sigma-Aldrich and were used without further purification. Phenylboronic acid catechol ester (**be**)^1^ and *trans*-pentafluorostilbazole (**pf-sbz**)^2^ were synthesized following reported procedures.

**Formation of be-pf-sbz apohost, solvates and cocrystal**. Synthesis of phenylboronic ester catechol adduct of *trans*-pentafluorostilbazole (**be-pf-sbz**) was adapted from a previously reported procedure.^3^ Specifically, phenylboronic ester (**be**) (30 mg, 0.1530 mmol) and *trans*-pentafluorostilbazole (**pf-sbz**) (41.5 mg, 0.1530 mmol) were dissolved in a *p­-*xylene (2 mL) and heated until the reddish solution became clear. Single crystals suitable for single crystal X-ray diffraction (scXRD) were observed after 2 days of slow evaporation.

Syntheses of solvates of phenylboronic ester catechol adduct of *trans*-pentafluorostilbazole **be-pf-sbz⊃guest** were achieved by dissolving **be** (30 mg, 0.1530 mmol) and **pf-sbz** (41.5 mg, 0.1530 mmol) in a suitable solvent (e.g., benzene, toluene and *o*-xylene, 2 mL) and heated until the reddish solution became clear. Single crystals of solvates **be-pf-sbz⊃ben**, **be-pf-sbz⊃tol**, **be-pf-sbz⊃*o*-xyl** suitable for scXRD were observed after 2 days of slow evaporation.

Synthesis of cocrystal with stilbene (**be-pf-sbz⊃sbn**) was carried out by dissolving **be** 30 mg, 0.153 mmol), **pf-sbz** (41.5 mg, 0.153 mmol) and **sbn** in acetonitrile (2 mL). Single crystals of **be-pf-sbz⊃sbn** suitable for scXRD were observed after 2 days of slow evaporation.

^1^H NMR spectra were recorded using a Bruker AVANCE 300 NMR spectrometer operating at 300 MHz using DMSO-*d*_6_ as NMR solvent.

# X-ray diffraction data

**X-ray instruments and methods**. Single crystal X-ray diffraction (scXRD) data of **be-pf-sbz**, **be-pf-sbz⊃ben** and **be-pf-sbz⊃tol** were collected on a Nonius KappaCCD single-crystal X-ray diffractometer; **be-pf-sbz⊃tol** and **be-pf-sbz⊃*o*-xyl** were collected on a Bruker Nonius APEX II Kappa single-crystal X-ray diffractometer using MoKα radiation (*λ* = 0.71073 Å), graphite monochromator. Structure solution, refinement and data output were carried out with the Olex2 software package using SHELXS-97 and SHELXL-2014 for the refinement.^4^ Non-hydrogen atoms were refined anisotropically and hydrogen atoms were placed in geometrically calculated positions using a riding model. Crystal structures were generated using Mercury.

**Supplementary Table 1.** Crystallographic parameters for **be-pf-sbz⊃ben**, **be-pf-sbz⊃tol** and **be-pf-sbz⊃*o*-xyl**.

| Compound name | **be-pf-sbz**⊃**ben** |  | **be-pf-sbz**⊃**tol** |  | **be-pf-sbz**⊃***o*-xyl** |
| --- | --- | --- | --- | --- | --- |
| Empirical formula | C_31_H_21_BF_5_NO_2_ |  | C_32_H_23_BF_5_NO_2_ |  | C_33_H_25_BF_5_NO_2_ |
| Formula weight | 545.30 |  | 559.32 |  | 573.35 |
| Temperature/K | 298.15 |  | 190.15 |  | 150.15 |
| Crystal system | monoclinic |  | orthorhombic |  | orthorhombic |
| Space group | P2_1_/n |  | P2_1_2_1_2_1_ |  | P2_1_2_1_2_1_ |
| a/Å | 6.6635(7) |  | 6.2113(6) |  | 6.2020(6) |
| b/Å | 13.7233(14) |  | 14.5523(15) |  | 14.6839(15) |
| c/Å | 29.079(3) |  | 29.338(3) |  | 29.639(3) |
| α/° | 90 |  | 90 |  | 90 |
| β/° | 91.353(5) |  | 90 |  | 90 |
| γ/° | 90 |  | 90 |  | 90 |
| Volume/Å^3^ | 2658.4(5) |  | 2651.8(5) |  | 2699.2(5) |
| Z | 4 |  | 4 |  | 4 |
| ρ_calc_g/cm^3^ | 1.362 |  | 1.401 |  | 1.411 |
| μ/mm^‑1^ | 0.108 |  | 0.110 |  | 0.110 |
| F(000) | 1120.0 |  | 1152.0 |  | 1184.0 |
| Crystal size/mm^3^ | 0.37 × 0.26 × 0.1 |  | 0.12 × 0.11 × 0.07 |  | 0.35 × 0.31 × 0.14 |
| Radiation | MoKα (λ = 0.71073) |  | MoKα (λ = 0.71073) |  | MoKα (λ = 0.71073) |
| 2Θ range for data collection/° | 2.802 to 50.692 |  | 5.768 to 52.836 |  | 4.97 to 52.914 |
| Index ranges | -7 ≤ h ≤ 8, -16 ≤ k ≤ 16, -34 ≤ l ≤ 34 |  | -7 ≤ h ≤ 7, -18 ≤ k ≤ 12, -36 ≤ l ≤ 35 |  | -7 ≤ h ≤ 7, -18 ≤ k ≤ 18, -37 ≤ l ≤ 34 |
| Reflections collected | 15174 |  | 34760 |  | 28726 |
| Independent reflections | 4823 [R_int_ = 0.0633, R_sigma_ = 0.0618] |  | 5420 [R_int_ = 0.0786, R_sigma_ = 0.0857] |  | 5550 [R_int_ = 0.0442, R_sigma_ = 0.0396] |
| Data/restraints/parameters | 4823/0/356 |  | 5420/45/370 |  | 5550/0/381 |
| Goodness-of-fit on F^2^ | 1.009 |  | 0.996 |  | 0.835 |
| Final R indexes [I>=2σ (I)] | R_1_ = 0.0490, wR_2_ = 0.0968 |  | R_1_ = 0.0466, wR_2_ = 0.0732 |  | R_1_ = 0.0367, wR_2_ = 0.1039 |
| Final R indexes [all data] | R_1_ = 0.1391, wR_2_ = 0.1263 |  | R_1_ = 0.1138, wR_2_ = 0.0894 |  | R_1_ = 0.0500, wR_2_ = 0.1184 |
| Largest diff. peak/hole / e Å^-3^ | 0.18/-0.16 |  | 0.19/-0.19 |  | 0.15/-0.15 |
| CCDC Number | 1943957 |  | 1943958 |  | 1943959 |

**Supplementary Table 2.** Crystallographic parameters for **be-pf-sbz⊃sbn** and **be-pf-sbz**.

| Compound name |  |  |  | **be-pf-sbz**⊃**sbn** |  |  | **be-pf-sbz** |
| --- | --- | --- | --- | --- | --- | --- | --- |
| Empirical formula |  |  |  | C_32_H_21_BF_5_NO_2_ |  |  | C_25_H_15_BF_5_NO_2_ |
| Formula weight |  |  |  | 557.31 |  |  | 467.19 |
| Temperature/K |  |  |  | 298.15 |  |  | 296.15 |
| Crystal system |  |  |  | monoclinic |  |  | monoclinic |
| Space group |  |  |  | P2_1_/n |  |  | P2_1_/c |
| a/Å |  |  |  | 6.2951(13) |  |  | 12.1231(12) |
| b/Å |  |  |  | 28.801(6) |  |  | 14.6648(15) |
| c/Å |  |  |  | 15.556(3) |  |  | 12.4002(12) |
| α/° |  |  |  | 90 |  |  | 90 |
| β/° |  |  |  | 90.72(3) |  |  | 102.287(5) |
| γ/° |  |  |  | 90 |  |  | 90 |
| Volume/Å^3^ |  |  |  | 2820.1(10) |  |  | 2154.0(4) |
| Z |  |  |  | 4 |  |  | 4 |
| ρ_calc_g/cm^3^ |  |  |  | 1.313 |  |  | 1.441 |
| μ/mm^‑1^ |  |  |  | 0.104 |  |  | 0.120 |
| F(000) |  |  |  | 1144.0 |  |  | 952.0 |
| Crystal size/mm^3^ |  |  |  | 0.39 × 0.28 × 0.12 |  |  | 0.2 × 0.16 × 0.04 |
| Radiation |  |  |  | MoKα (λ = 0.71073) |  |  | MoKα (λ = 0.71073) |
| 2Θ range for data collection/° |  |  |  | 4.986 to 49.982 |  |  | 4.42 to 53.488 |
| Index ranges |  |  |  | -7 ≤ h ≤ 7, -31 ≤ k ≤ 32, -18 ≤ l ≤ 17 |  |  | -14 ≤ h ≤ 15, -18 ≤ k ≤ 18, -15 ≤ l ≤ 15 |
| Reflections collected |  |  |  | 14509 |  |  | 12709 |
| Independent reflections |  |  |  | 4739 [R_int_ = 0.0357, R_sigma_ = 0.0363] |  |  | 4563 [R_int_ = 0.0740, R_sigma_ = 0.0915] |
| Data/restraints/parameters |  |  |  | 4739/36/369 |  |  | 4563/0/307 |
| Goodness-of-fit on F^2^ |  |  |  | 1.038 |  |  | 0.939 |
| Final R indexes [I>=2σ (I)] |  |  |  | R_1_ = 0.0494, wR_2_ = 0.1194 |  |  | R_1_ = 0.0590, wR_2_ = 0.1424 |
| Final R indexes [all data] |  |  |  | R_1_ = 0.0839, wR_2_ = 0.1377 |  |  | R_1_ = 0.1670, wR_2_ = 0.1910 |
| Largest diff. peak/hole / e Å^-3^ |  |  |  | 0.35/-0.20 |  |  | 0.24/-0.18 |
| CCDC Number |  |  |  | 1943960 |  |  | 1943956 |
|  |  |  |  |  |  |  |  |

**Supplementary Table 3.** Selected supramolecular interactions for **be-pf-sbz**⊃**ben**, **be-pf-sbz**⊃**tol**, **be-pf-sbz**⊃***o*-xyl**, **be-pf-sbz**⊃**sbn** and **be-pf-sbz**.

| Crystal | D−H⋅⋅⋅A/  Centroid⋅⋅⋅Centroid | *d*(D−H)  [Å] | *d*(H⋅⋅⋅A)  [Å] | *d*(D⋅⋅⋅A)  [Å] | *∠*(D−H⋅⋅⋅A)  [deg] | Symmetry code |
| --- | --- | --- | --- | --- | --- | --- |
| **be-pf-sbz**⊃**ben** | C26B−H26B⋅⋅⋅F4 | 0.93 | 2.603 | 3.444(2) | 150.7 | 1+x, y, z |
|  | C12−H12⋅⋅⋅F1 | 0.93 | 2.66 | 3.496(3) | 137.0 | 5/2-x, 1/2+y, 3/2-z |
|  | C9−H9⋅⋅⋅F3 | 0.93 | 2.69 | 3.434(3) | 138.1 | -1/2+x, 1/2-y, 1/2+z |
|  | Cg1⋅⋅⋅Cg2^[a]^ | - | - | 3.745 | - | x, y, z |
|  | Cg1⋅⋅⋅Cg3^[a]^ | - | - | 3.706 | - | 3/2-x, -1/2+y, 3/2-z |
| **be-pf-sbz**⊃**tol** | C4−H4⋅⋅⋅O2 | 0.95 | 2.50 | 3.411(4) | 161.6 | 2-x, 1/2+y, 3/2-z |
|  | C11−H11⋅⋅⋅F3 | 0.95 | 2.53 | 3.303(4) | 138.3 | 1/2-x, -y, 1/2+z |
|  | C30A−H30A⋅⋅⋅F2 | 0.95 | 2.60 | 3.465(2) | 159.5 | -1+x, y, z |
|  | C2−H2⋅⋅⋅O1 | 0.95 | 2.62 | 3.347(4) | 133.2 | 1+x, y, z |
|  | Cg4⋅⋅⋅Cg5^[b]^ | - | - | 3.790 | - | x, y, z |
|  | Cg4⋅⋅⋅Cg6^[b]^ | - | - | 3.669 | - | 1-x, -1/2+y, 3/2-z |
|  | C32A−H32A⋅⋅⋅Cg7^[b]^ | 0.98 | 2.87 | 3.827 | 157.0 | 1/2+x, 1/2-y, 1-z |
| **be-pf-sbz**⊃***o*-xyl** | C33−H33⋅⋅⋅F3 | 0.98 | 2.59 | 3.350(3) | 134.2 | x, y, z |
|  | C32−H32⋅⋅⋅F2 | 0.98 | 2.87 | 3.548(3) | 126.6 | x, y, z |
|  | C33−H33⋅⋅⋅F2 | 0.98 | 2.59 | 3.534(3) | 162.1 | -1+x, y, z |
|  | C11−H11⋅⋅⋅F3 | 0.95 | 2.56 | 3.376(3) | 143.5 | 1/2-x, 1-y, 1/2+z |
|  | C6−H6⋅⋅⋅O1 | 0.95 | 2.62 | 3.397(3) | 137.8 | 1+x, y, z |
|  | C4−H4⋅⋅⋅O2 | 0.95 | 2.45 | 3.398(3) | 162.7 | 2-x, -1/2+y, -z |
|  | C12−H12⋅⋅⋅O2 | 0.95 | 2.80 | 5.525(3) | 133.6 | -1+x, y, z |
|  | Cg8⋅⋅⋅Cg9^[c]^ | - | - | 4.129 | - | x, y, z |
|  | Cg8⋅⋅⋅Cg10^[c]^ | - | - | 3.642 | - | 1-x, 1/2+y, 3/2-z |
|  | C3−H3⋅⋅⋅Cg10^[c]^ | 0.95 | 2.754 | 2.754(1) | 12.4 | 1-x, -1/2+y, 3/2-z |
| **be-pf-sbz**⊃**sbn** | C31A−H31A⋅⋅⋅F2 | 0.93 | 2.51 | 3.312(2) | 144.9 | x, y, z |
|  | C4−H4⋅⋅⋅F4 | 0.93 | 2.74 | 3.465(3) | 135.2 | -1/2-x, -1/2+y, 1-2-z |
|  | C3−H3⋅⋅⋅F3 | 0.93 | 2.87 | 3.789(3) | 170.9 | -1/2-x, -1/2+y, 1-2-z |
|  | C5−H5⋅⋅⋅F4 | 0.93 | 2.94 | 3.824(3) | 158.6 | 1/2+x, 1/2+y, 1-2+z |
|  | C13−H13⋅⋅⋅O1 | 0.93 | 2.977 | 3.840(3) | 154.8 | 1+x, y, z |
|  | Cg11⋅⋅⋅Cg12^[d]^ | - | - | 4.013 | - | -1+x, y, z |
|  | Cg11⋅⋅⋅Cg13^[d]^ | - | - | 3.763 | - | 1/2+x, 1/2-y, -1/2+z |
|  | C29A−H29A⋅⋅⋅Cg14^[d]^ | 0.93 | 2.900 | 3.775(3) | 17.4 | 1-x, 1-y, 1-z |
| **be-pf-sbz** | C14−H14⋅⋅⋅F5 | 0.93 | 2.46 | 3.352(3) | 135.2 | x, 3/2-y, z |
|  | C19−H19⋅⋅⋅F5 | 0.93 | 2.57 | 3.481(3) | 167.8 | x, 3/2-y, z |
|  | C13−H13⋅⋅⋅O1 | 0.93 | 2.70 | 3.605(3) | 163.6 | 1-x, 1-y, -z |
|  | Cg15⋅⋅⋅Cg16^[e]^ | - | - | 3.676 | - | 1-x, 1/2+y, 1/2-z |

^[a]^ Cg1 = C20−C25; Cg2 = C26B−C31B; Cg3 = C7−C12. ^[b]^ Cg4 = C20−C25; Cg5 = C26A−C31A; Cg6 = C7−C12; Cg7 = C1−C6. ^[c]^ Cg8 = C20−C25; Cg9 = C26−C31; Cg10 = C7−C12. ^[d]^ Cg11 = C20−C25; Cg12 = C26A−C31A; Cg13 = C1−C6; Cg14 = C7−C12 ^[e]^ Cg15 = C20−C25; Cg16 = C7−C12.

**
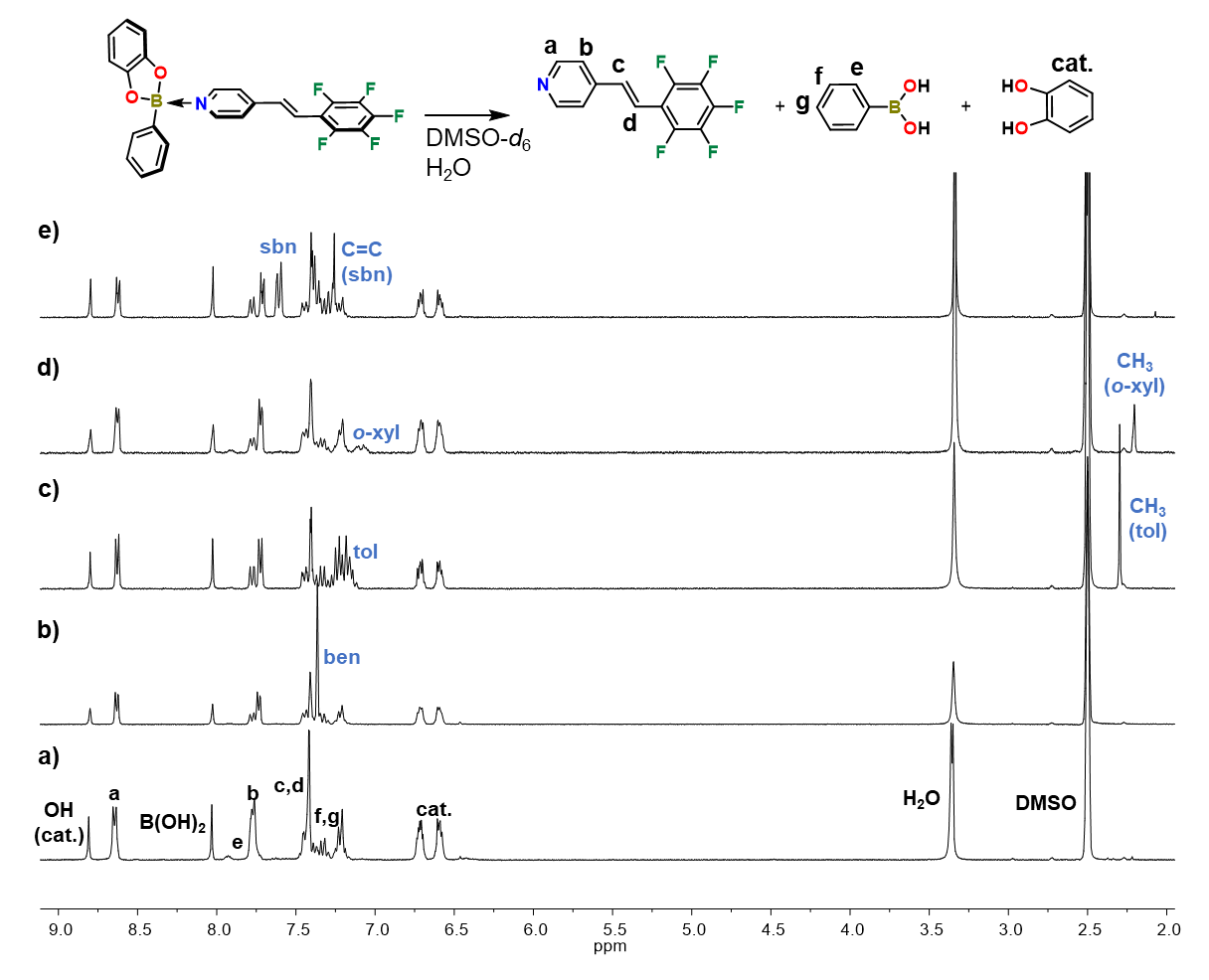
**

**Supplementary Figure 1.** ^1^H NMR spectra of single crystals of (a) **be-pf-sbz**, (b) **be-pf-sbz**⊃**ben**, (c) **be-pf-sbz**⊃**tol**, (d) **be-pf-sbz**⊃***o*-xyl** and (e) **be-pf-sbz**⊃**sbn** (300 MHz, DMSO-*d*_6_).

# References

1. Cruz-Huerta, J.; Salazar-Mendoza, D.; Hernández-Paredes, J.; Ahuactzi, I. F. H.; Höpfl, H., N-containing boronic esters as self-complementary building blocks for the assembly of 2D and 3D molecular networks. *Chem. Commun.* **2012,** *48* (35), 4241-4243.

2. Lorance, E. D.; Kramer, W. H.; Gould, I. R., Kinetics of reductive N− O bond fragmentation: the role of a conical intersection. *J. Am. Chem. Soc.* **2002,** *124* (51), 15225-15238.

3. Campillo-Alvarado, G.; D'mello, K. P.; Swenson, D. C.; Santhana Mariappan, S. V.; Höpfl, H.; Morales-Rojas, H.; MacGillivray, L. R., Exploiting Boron Coordination: B←N Bond Supports a [2+2] Photodimerization in the Solid State and Generation of a Diboron Bis-Tweezer for Benzene/Thiophene Separation. *Angew. Chem. Int. Ed.* **2019,** *58* (16), 5413-5416.

4. (a) Dolomanov, O. V.; Bourhis, L. J.; Gildea, R. J.; Howard, J. A.; Puschmann, H., OLEX2: a complete structure solution, refinement and analysis program. *J. Appl. Crystallogr.* **2009,** *42* (2), 339-341; (b) Sheldrick, G. M., Crystal structure refinement with SHELXL. *Acta Cryst. C* **2015,** *71* (1), 3-8; (c) Sheldrick, G. M., SHELXS-97, Program for crystal structure solution. University of Göttingen, Germany Göttingen: 1997.
